# Spontaneous Eosinophilic Nasal Inflammation in a Genetically-Mutant Mouse: Comparative Study with an Allergic Inflammation Model

**DOI:** 10.1371/journal.pone.0035114

**Published:** 2012-04-11

**Authors:** Seok Hyun Cho, Sun Young Oh, Zhou Zhu, Joan Lee, Andrew P. Lane

**Affiliations:** 1 Department of Otolaryngology-Head and Neck Surgery, Johns Hopkins University School of Medicine, Baltimore, Maryland, United States of America; 2 Department of Otorhinolaryngology-Head and Neck Surgery, College of Medicine, Hanyang University, Seoul, Korea; 3 Division of Allergy and Clinical Immunology, Johns Hopkins University School of Medicine, Baltimore, Maryland, United States of America; Instituto de Biofisica Carlos Chagas Filho, Universidade Federal do Rio de Janeiro, Brazil

## Abstract

**Background:**

Eosinophilic inflammation is a hallmark of chronic rhinosinusitis with nasal polyps. To model this disease process experimentally, nasal sensitization of mice with ovalbumin or aspergillus has been described. Here, we describe a genetically mutant mouse that develops robust spontaneous nasal eosinophilic inflammation. These mice lack the enzyme SHP-1 that down-regulates the IL-4Rα/stat6 signaling pathway. We compared nasal inflammation and inflammatory mediators in SHP-1 deficient mice (*mev*) and an ovalbumin-induced nasal allergy model.

**Methods:**

A novel technique of trans-pharyngeal nasal lavage was developed to obtain samples of inflammatory cells from the nasal passages of allergic and *mev* mice. Total and differential cell counts were performed on cytospin preparations. Expression of tissue mRNA for IL-4, IL-13, and mouse beta-defensin-1 (MBD-1) was determined by quantitative PCR. Eotaxin in the lavage fluid was assessed by ELISA.

**Results:**

Allergic and *mev* mice had increased total cells and eosinophils compared with controls. Expression of IL-4 was similarly increased in both allergic and *mev* mice, but expression of IL-13 and eotaxin was significantly greater in the allergic mice than *mev* mice. Eotaxin was significantly up-regulated in both allergic rhinitis and *mev* mice. In both models of eosinophilic inflammation, down-regulation of the innate immune marker MBD-1 was observed.

**Conclusions:**

The *mev* mice display spontaneous chronic nasal eosinophilic inflammation with potential utility for chronic rhinosinusitis with nasal polyps research. The eosinophilic infiltrate is more robust in the *mev* mice than allergic mice, but Th2 cytokine expression is not as pronounced. Decreased MBD-1 expression in both models supports the concept that Th2-cytokines down-regulate sinonasal innate immunity in humans, and suggests a role for mouse models in investigating the interaction between adaptive and innate immunity in the sinonasal mucosa.

## Introduction

A defining characteristic of chronic rhinosinusitis with nasal polyps (CRSwNP) is the presence of an eosinophilic inflammatory infiltrate and a Th2 cytokine predominance [1]. The initiating cause of CRSwNP is unknown, and the pathologic processes that underlie the persistent inflammation are poorly understood. In order to study the cellular and molecular basis of CRSwNP, model systems have been described in vitro and in animal species, including rodents and sheep [2], [3]. While mouse sinuses lack the size and anatomic features of human sinuses, these model systems have the significant advantage of powerful genetic tools available to manipulate gene expression experimentally. In recent years, allergen challenge models have been created that result in chronic eosinophilic rhinosinusitis in mice [4]. However, no transgenic mouse models have been utilized to explore genetically-driven Th2-biased sinonasal inflammation.

The sinonasal mucosal immune system plays a critical role in protecting the host from potential pathogens that enter the airway during breathing. Sinonasal epithelial cells act as sentinels and early responders, detecting pathogen-associated molecules via pattern-recognition receptors and elaborating a variety of antimicrobial effectors [5], [6]. They interact bi-directionally with intra-epithelial lymphocytes and other adaptive immune elements in order to coordinate an antimicrobial defense. While the underlying pathophysiology of CRSwNP remains under investigation, gene expression analysis suggests that a number of proteins involved in innate immunity, barrier function, and repair are down-regulated in diseased sinus mucosa [7]. Whether this alteration represents a cause or an effect of chronic inflammation remains to be elucidated. There is evidence that Th2 cytokines directly suppress epithelial cell innate immune function in-vitro, perhaps implying that the adaptive immune system mediates the observed innate immune deficiency in CRSwNP [8], [9]. Studies of cellular and molecular mechanisms are difficult or impractical to carry out in human patients, and in vitro models utilizing human tissue have significant limitations. Thus, animal models of sinonasal inflammation provide a critical opportunity to examine the interplay between the innate and adaptive immune systems in great depth.

Models of nasal allergic inflammation in the mouse support the concept that Th2-mediated inflammation diminishes effective anti-bacterial immunity in the airway. For example, Naclerio et al. showed that mice with allergic rhinitis had a worse course of acute sinusitis when inoculated with *Streptococcus pneumonia* [10]. Ongoing allergic provocation resulted in an increased percentage of sinuses with neutrophil clusters and more *S. pneumoniae* colonies at the time of sacrifice. In addition, Beisswenger et al. used an allergic mouse model to study pulmonary antimicrobial innate immune defense [11]. Sensitized allergic mice inoculated with *Pseudomonas aeruginosa* had a greater number of viable bacteria in the lung 24 hours after infection when compared to non-sensitized animals. In preliminary studies, we have demonstrated reduced innate immune gene expression in a nasally-sensitized ovalbumin allergy model. While allergic rhinosinusitis mouse models do achieve a localized Th2-skewed immune response, the degree and persistence of eosinophilic inflammation does not closely approximate that observed in CRSwNP.

Src homology 2 domain-containing protein tyrosine phosphatase (SHP-1) is a negative regulator of the IL-4Rα signaling pathway. Once phosphorylated and activated, SHP-1 binds to and dephosphorylates its target molecules to terminate signaling [12]. A genetically-altered mouse strain known as motheaten viable (*mev*) has been found that is deficient in SHP-1. This mouse develops an asthma-like phenotype in the lung with a very prominent eosinophilic infiltrate [13], [14]. While the lower airway has been studied in detail, the phenotype of the upper airway of *mev* mice has not been reported. In this paper, we characterized nasal inflammation in this mutant mouse, and compare its features to the commonly-utilized allergen sensitization model. It is hoped that detailed study of eosinophilic sinonasal inflammation in mice will permit insights into human CRSwNP that will direct future investigation and novel therapeutic strategies.

## Materials and Methods

### Animals

Female BALB/c and *mev* (Ptpn^6mev^) mice (6–8 weeks old) were obtained from the Jackson Laboratory (Bar Harbor, Maine). Heterozygous mice (Ptpn^6mev/+^, C57BL/6 background) were interbred to generate WT, heterozygous, and homozygous mice. In this study, we used *mev* homozygous mice (n = 12) as a spontaneous eosinophilic rhinitis model and WT littermates (n = 11) as untreated controls. BALB/c mice were used for PBS controls (n = 13) and OVA-induced allergic rhinitis (n = 11). Animals were housed in a specific pathogen-free facility with single ventilated cages and received OVA-free diet. All animal experiments were approved by the Johns Hopkins Institutional Animal Care and Use Committee.

### Allergen sensitization and challenge

**Figure 1:**
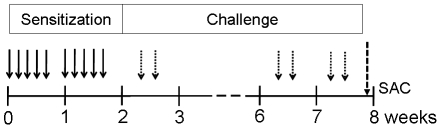
Experimental design used to study the effect of nasal exposure to ovalbumin in mice. BALB/c mice (11–13 per group) were sensitized by intranasal administration of 100 µg of ovalbumin in 20 µl PBS in the absence of adjuvants 5 times a week for 2 weeks. After that, mice were challenged by intranasal administration of same dose of ovalbumin two times a week for 6 weeks. As a control, mice were treated with PBS with the same protocol. Two days after the last challenge, mice were sacrificed for obtaining the NAL fluids and tissue samples.

Ovalbumin (OVA, grade V; Sigma Chemical Co, St Louis, Mo) was used for nasal sensitization of BALB/c mice. The mice were positioned supine and maintained in an awake state without use of sedatives. For the first two weeks, mice were sensitized five time per week by local intranasal instillations of 100 µg of OVA in 20 µl of PBS without alum (Al(OH)_3_). After sensitization, from day 15 to 56, mice were challenged intranasally two times per week (total 12 times) with the same OVA preparation (Figure 1). Control groups were challenged with equivalent volumes of PBS.

### Trans-pharyngeal nasal lavage and cytology

**Figure 2:**
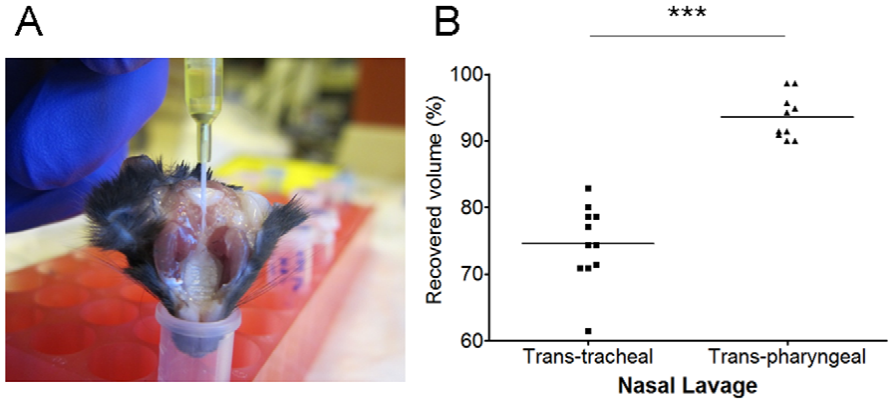
The method of NAL technique used in this study. (A) Photograph of the trans-pharyngeal approach to obtain nasal lavage samples from mouse models. (B) Comparison of yields in the recovered fluid volume after the NAL between trans-tracheal and trans-pharyngeal approaches (***p<0.001).

All samples were collected 48 hours after the last allergen challenge. After mice were anesthetized, the left ventricle was cannulated and systemic blood was cleared by perfusion with cold PBS. To obtain nasal airway lavage (NAL) samples, a trans-pharyngeal modification of the previously described trans-tracheal technique [15] was developed. The upper airway, including palatopharyngeal region, was isolated by scissor dissection, and the mouse head was separated at the level of the larynx. A 24G IV catheter was inserted through the pharyngeal opening into the choana (Figure 2A). Two consecutive volumes of 350 µl of PBS were instilled, and the fluid was collected from the nostrils. NAL fluids were centrifuged, and supernatants were stored at −80°C until assayed. The cell pellet was resuspended with 100 µl PBS and the total number of cells was determined. Cytospin slides were prepared and stained with Diff-Quik stain (Dade Behring-Switzerland), followed by differential count of at least 200 cells per slide. The yield of trans-pharyngeal NAL was directly compared to trans-tracheal technique, demonstrating a significantly increased volume of recovered fluid with the trans-pharyngeal method (93.4%±1.05 versus 74.6%±1.78 with the trans-tracheal method, P = 0.0001, Figure 2B).

### Harvesting of nasal mucosa and histology

To harvest nasal tissues for mRNA extraction, the skull was bisected sagittally, and septal and turbinate mucosa were removed and placed in 1 ml of RNAlater solution (Ambion, Inc). The tissue was stored in 4°C until RNA extraction. For nasal histology, mouse heads were fixed with paraformaldehyde and decalcified for 2 days before embedment in paraffin. Coronal paraffin sections were cut on a microtome and stained with hematoxylin and eosin (H&E). Images were acquired using the Zeiss Axio Imager.A2 microscope and measurements made using the Axiovision 4.8 software (Carl Zeiss Micro-imaging, Thornwood, NJ). The number of infiltrated eosinophils was counted on respiratory turbinate sections at five random high power fields (HPF, 40×) and calculated mean values for comparison. To demonstrate mucosal remodeling, epithelial thickness was measured at the same respiratory turbinate locations, from the basement membrane to the nasal lumen.

### RNA extraction and reverse transcription

Total RNA was isolated with the RNeasy Mini kit (Qiagen, Valencia, CA) using the manufacturer's protocol. RNA was quantified spectrophotometrically and absorbance ratios at 260/280 nm were >1.80 for all samples studied. Five hundred nanograms of total RNA was reverse transcribed in a 20 µl volume with random hexamer primers (Invitrogen, Carlsbad, CA), 20 U of RNase inhibitor (Applied Biosystems, Foster City, CA), and the Omniscript RT kit (Qiagen) under conditions provided by the manufacturer.

### Real-time polymerase chain reaction

Real-time PCR was performed using the Applied Biosystems StepOnePlus machine (Foster City, CA) under standard cycling parameters. The Taqman gene expression system (Applied Biosystems) was used to measure mRNA levels of IL-4, IL-13, and mouse β-defensin-1 (MBD-1). The reaction mix consisted of 0.5 µg total RNA for target genes or 0.05 ng total RNA for GAPDH, 10 µl of TaqMan Fast Universal PCR Master Mix, 1.5–5 mol/L probe target genes or 1.0 mol/L probe GAPDH, in a total volume of 20 µl. Each PCR run was accompanied by housekeeping gene GAPDH as an internal control. Amplicon expression in each sample was normalized to its GAPDH RNA content and the level of expression of target mRNA was determined as the delta C_t_, the difference in threshold cycles for each target and housekeeping gene. Relative gene expression was calculated by the 2^−ΔCt^.

### Measurement of eotaxin in the NAL fluids

Standard antibody-based ELISAs were used to measure concentrations of eotaxin (polyclonal mouse anti-eotaxin, R&D systems) in the NAL fluid samples. The limit of detection for the eotaxin ELISA kit was 3.0 pg/ml.

### Statistical analysis

All values are shown as mean ± standard deviation of the mean. Total cell and differential cells count in three groups were compared with two-way ANOVA test. The expression of inflammatory mediator mRNA between genetically matched groups was compared with non-parametric test (Mann-Whitney test). Statistical significance was considered to be *P*<.05. Results were analyzed with the use of the statistical software Prism 4 (GraphPad, Inc).

## Results

### Increased total cells and eosinophils in NAL fluids in allergic rhinitis and *mev* mice

**Figure 3:**
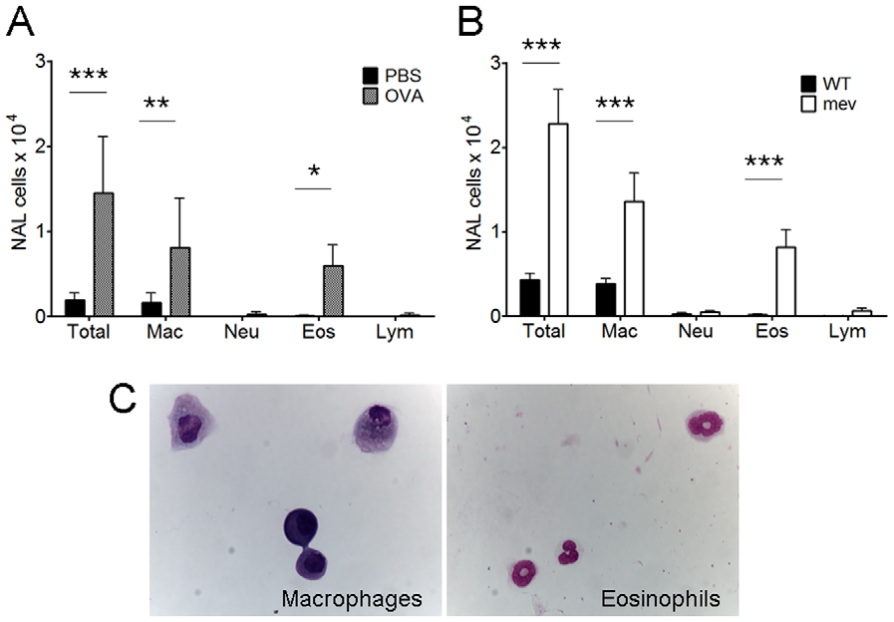
Cytology in the NAL fluids of allergic rhinitis and *mev* mice. (A and B) Total and differential cell counts in the NAL fluids collected from controls, allergic rhinitis (OVA) and *mev* mice (*p<0.05, **p<0.01, and ***p<0.001). (C) Representative photos showing macrophages and eosinophils (Diff-Quick stain, 40×).

Because the OVA-induced allergic rhinitis model was induced in BALB/c background mice, comparisons with PBS controls were made in BALB/c mice. The *mev* mice were generated in C57BL/6 background and were therefore compared with untreated C57BL/6 mice. The total cell numbers in the NAL was significantly increased in both allergic rhinitis (Figure 3A. 1.45×10^4^ cells, P<0.0001) and *mev* mice (Figure 3B. 2.28×10^4^ cells, P<0.0001) compared with their respective controls. Differential cell counts of the NAL fluids were performed on cytospins using morphology criteria (Figure 3C). The number of eosinophils in the NAL was significantly increased in both allergic rhinitis (5.94×10^3^ cells, P<0.05) and *mev* mice (8.19×10^3^ cells, P<0.0001) to similar levels. The percentage of macrophages was also significantly increased in both allergic rhinitis (P<0.001) and *mev* mice (P<0.0001) compared to controls.

### Tissue eosinophilia is induced by OVA challenge and SHP-1 enzyme deficiency

**Figure 4:**
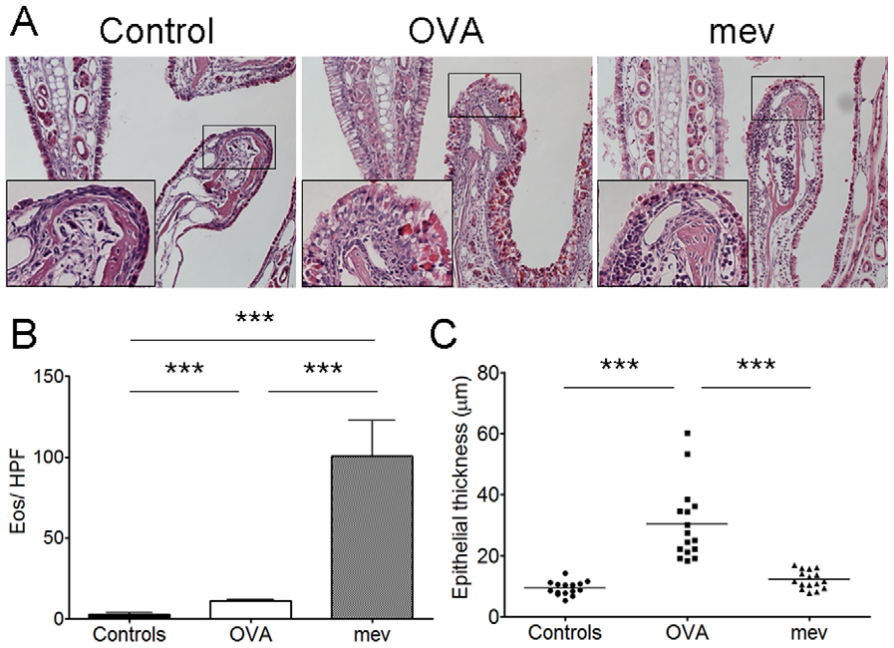
Histology of allergic rhinitis and *mev* mice. (A) Hematoxylin and eosin stains of the nasal cavity from controls, allergic rhinitis (OVA) and *mev* mice. (B) In allergic and *mev* mice, there is clearly increased nasal inflammation with eosinophilia, as compared to controls. (C) Epithelial thickness significantly increased in allergic mice compared with controls and *mev* mice (*p<0.05, **p<0.01, and ***p<0.001).

We compared hematoxylin and eosin stains of the nasal cavity from WT, allergic rhinitis mouse model and *mev* mice (Figure 4A). In untreated C57BL/6 and PBS treated BALB/c mice, there was no evidence of nasal inflammation and epithelial change. However, in allergic rhinitis and *mev* mice, there was severe tissue inflammation with eosinophilia. The number of infiltrating eosinophils per HPF was significantly increased in allergic rhinitis (P<0.0001) and *mev* mice (P<0.0001) compared with controls (Figure 4B). Compared with the epithelium of controls and *mev* mice, the turbinate epithelium of OVA-induced allergic rhinitis was significantly thickened (P<0.0001, Figure 4C). However, there was no definite evidence of epithelial remodeling in *mev* mice compared with controls.

### Expression of Th2 cytokines in allergic rhinitis and *mev* mice

**Figure 5:**
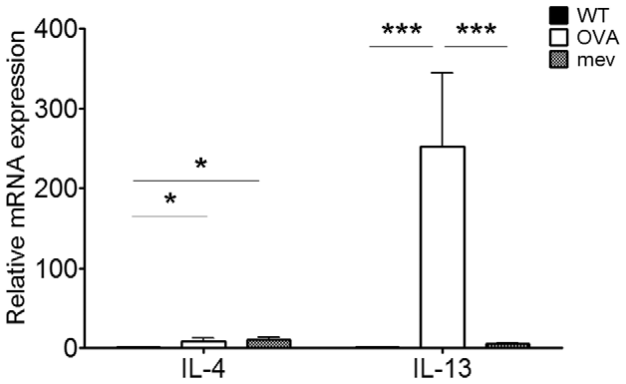
Relative mRNA expression of Th2 cytokines (IL-4 and IL-13) in controls, allergic rhinitis (OVA), and *mev* mice (*p<0.05 and ***p<0.001). IL-4 is significantly up-regulated in both allergic rhinitis and *mev* mice compared with controls. However, IL-13 significantly increased only in OVA model.

mRNA expression of the Th2 cytokines IL-4 and IL-13 was assessed in the nasal mucosal samples and compared between the genetically matched groups (Figure 5). IL-4 significantly increased in both allergic rhinitis (8.34 fold, P = 0.0424) and *mev* mice (11.16 fold, P = 0.024) compared with respective controls. IL-13 was significantly increased in allergic rhinitis (252.67 fold, P = 0.0001) but not in *mev* mice.

### Increased eotaxin in the NAL fluids of allergic rhinitis and *mev* mice

**Figure 6:**
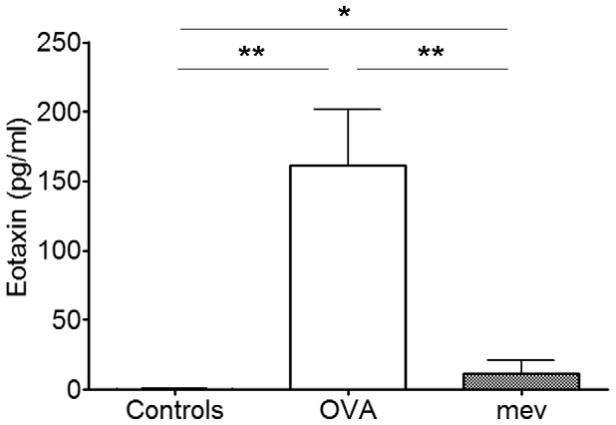
Eotaxin expression in the NAL fluids of controls, allergic rhinitis (OVA) and *mev* mice. Eotaxin significantly increased in both OVA model and *mev* mice compared to controls while it was significantly higher in OVA model than *mev* mice (*p<0.05 and **p<0.01).

The levels of eotaxin in the NAL fluids were measured by ELISA (Figure 6). Eotaxin was significantly up-regulated in both allergic rhinitis (161.4 pg/ml±40.16, P = 0.0036) and *mev* mice (11.9 pg/ml±9.53, P = 0.04) when compared with controls. Eotaxin in the NAL fluids showed same basal level of expression in controls of BALB/c and C57BL/6 mice.

### Decreased expression of MBD-1 in allergic rhinitis and *mev* mice

**Figure 7:**
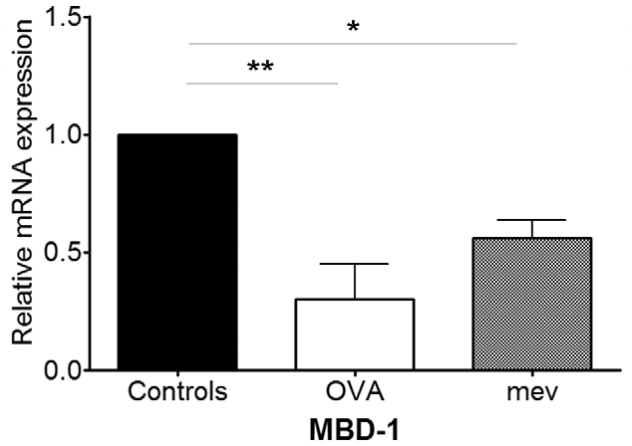
Relative mRNA expression of MBD-1 in controls, allergic rhinitis (OVA), and *mev* mice (*p<0.05 and **p<0.01). Expression of MBD-1 significantly decreased in both OVA model and *mev* mice.

The mRNA expression of MBD-1 significantly decreased in both allergic rhinitis (3.44 fold, P = 0.0242) and *mev* mice (1.79 fold, P = 0.0182, Figure 7). There was no difference in MBD-1 expression between them.

## Discussion

Animal models provide an important experimental platform for studying pathophysiologic mechanisms in CRS. Mice are a particularly attractive system for model development because they are genetically homogeneous and there is a large body of information present in the literature about the cellular, biochemical, molecular biological aspects of inflammation in this species. Furthermore, mice are the only practical species in which transgenic animals can be generated, and specific targeted mutations in existing mouse strains are available. One of the central characteristics of human CRSwNP is the presence of profound Th2-skewed sinonasal eosinophilic inflammation [1]. While certain aspects of this inflammatory state may be mimicked in a mouse that is sensitized and intranasally challenged with antigen for an extended period of time, the development of genetically-modified mice with spontaneous eosinophilic inflammation may afford new insights that are complementary and extend our understanding.

As mouse models are generated and optimized to the study of CRS, it is critical to establish outcome measures that quantify the inflammatory state and response to intervention. The novel nasal lavage technique described in this paper is simple to perform, inexpensive, and shows cell count results that mirror histological features. Most importantly, our NAL techniques showed an excellent yield having increased returned fluid volume after NAL and remarkable consistency among different samples when compared with the more conventional trans-tracheal NAL method (Figure 2). This difference is likely due to leakage of trans-tracheal lavage fluid through the oral cavity. The analysis of cell counts and mediator levels using this novel trans-pharyngeal NAL provides a broadly applicable tool to characterize the severity and type of inflammatory responses in mouse models of sinonasal disease.

There is an extensive literature regarding allergen-induced lower and upper airway inflammation models in mice. In most cases, the inflammation is generated by systemic sensitization through the intraperitoneal or subcutaneous route with subsequent local challenge with nebulized or intranasal allergen [16], [17]. In the present study, we have successfully created a similar model using 8 weeks of intranasal OVA instillation without systemic injection or use of any adjuvant such as alum. We observe a robust eosinophilic tissue infiltrate, increased mRNA expression of Th2 cytokines IL-4 and IL-13 and protein expression of eotaxin in OVA mouse model. It is important to recognize that although mouse rhinosinusitis models have been previously described using fungal extracts for sensitization, the resulting eosinophilic inflammatory response does not appear to be dependent on the antigen [18]. In other words, there is no evidence that sinonasal inflammation in the mouse triggered by aspergillus better models features of human CRS (fungal-related or otherwise) than does ovalbumin or another antigen.

Strain-specific phenotypic differences between BALB/c and C57BL/6 mice are well known in asthma and allergic rhinitis models, with differences described in pro-inflammatory cytokines and inflammatory cells [19], [20]. BALB/c mice are Th2 prone, while C57BL/6 mice favor Th1 inflammation. Preliminary attempts to generate OVA-induced allergic rhinitis in the C57BL/6 mice did not result in reliably robust responses (data not shown). Therefore, we studied OVA-induced allergic rhinitis and *mev* mice with the use of strain-matched controls.

In *mev* mouse line, the deficiency of SHP-1 enzyme, results in IL-4 receptor over-activity with spontaneous inflammation developing in multiple organs, including, most prominently, the lung [19], [20], [21]. The pulmonary inflammation is Th2-skewed, evidenced by increased eosinophils in bronchoalveolar lavage, mucous metaplasia, epithelial hypertrophy, and subepithelial and parenchymal fibrosis in the lung tissue [13], [14]. At the molecular level, Th2 cytokines and chemokines, as well as phosphorylated STAT6, were significantly upregulated in the lungs of *mev* mice as compared with WT mice. Using a targeted gene deletion approach, the pulmonary inflammation in *mev* mice showed IL-13, IL-4Rα, and STAT6-dependence in the lung [13]. In the present study, we demonstrate that deficiency of SHP-1 also results in a sinonasal phenotype, characterized by eosinophilic rhinosinusitis comparable to the OVA-induced allergic rhinitis mouse model. In future studies, the downstream signaling pathways modulated by SHP-1, including IL-4Rα/STAT6 and phosphatidylinositol 3-kinase (PI3K) will be investigated in the *mev* mouse, to elucidate the interplay between Th2 cytokine signaling and innate immunity, barrier function, and repair.

While the innate and adaptive immunity represent two distinct arms of host defense, studies with human tissue suggest that a complex interaction exists between the two that may be dysregulated in CRS [7], [9]. Genetically mutant mouse models such as *mev* mice allow specific features of CRSwNP to be mimicked in an *in vivo* situation, in order to begin to dissect some of these critical relationships experimentally in a way that is not possible in human subjects or in cell or tissue explants culture systems. The comparison between the *mev* and the allergic rhinosinusitis mouse models demonstrates similarity in the overall eosinophilic inflammatory picture, but significant differences in the expression of the Th2-associated mediators IL-13 and eotaxin. In the lungs of *mev* mice there is a robust eosinophilia in bronchoalveolar lavage and the parenchyma with strong evidence of Th2-skewed inflammation [13], [14]. Although the nasal cavity also displays eosinophilic inflammation in the NAL fluids and mucosa, evidence for a Th2 inflammatory response is not as pronounced. Further studies are needed to determine the molecular mechanism underlying the spontaneous eosinophilic inflammation in the sinonasal tract. Interestingly, in comparison to the distinct increase in epithelial thickness seen in the OVA allergy model, *mev* mice maintain a relatively normal appearance of the nasal epithelium, despite the intense sub-epithelial eosinophilic infiltrate (Figure 4). This suggests that the response of the epithelium to Th2 inflammation may be shaped by other endogenous and exogenous signals that contribute to mucosal homeostasis.

In both models, expression of the innate antimicrobial MBD-1 is reduced, which is consistent with mRNA findings in human CRSwNP tissue and sinonasal epithelial cells in vitro [22]. MBD-1, a homolog of hBD-1, has antibacterial effects on gram-positive and -negative bacteria and has been detected in bronchial epithelial cells [23]. To our knowledge, the present study is the first report on MBD-1 expression in either allergic rhinosinusitis mouse models or *mev* mice. Further exploration in these mouse models is warranted to investigate the interaction between Th2 cytokines and epithelial cell innate immune function. The differential expression of inflammatory mediators between models may allow the roles of individual cytokines to be dissected.

Because current medical and surgical interventions fail to control CRSwNP effectively in the long-term, the condition can be frustrating for patients and otolaryngologists alike [24]. At this time, a significant obstacle to developing new therapeutic strategies for CRSwNP is the absence of animal models that allow study of the disease at the cellular and molecular level. CRSwNP is a complex multifactorial condition, and it is unlikely that a single animal model can reproduce all of the functional and morphological features of the human disease state. However, transgenic mouse strains can be created to mimic specific features of the disorder, and by utilizing the wide array of specific reagents available to dissect subcellular pathways of immunity and inflammation, a much greater appreciation of critical underlying mechanisms can be achieved. Thus, while larger animal species have proven useful to model sinus infection and endoscopic surgery, rigorous scientific investigation of inflammatory pathways is better suited to mouse models. The *mev* mouse and other transgenic and knockout strains either currently available or in development have great potential to tremendously advance the field of sinonasal mucosal immunology, with direct implications for understanding chronic inflammation in CRSwNP.
